# Specific immunosuppressive role of nanodrugs targeting calcineurin in innate myeloid cells

**DOI:** 10.1016/j.isci.2022.105042

**Published:** 2022-08-30

**Authors:** Miriam Colombo, Laura Marongiu, Francesca Mingozzi, Roberta Marzi, Clara Cigni, Fabio Alessandro Facchini, Rany Rotem, Mihai Valache, Giulia Stucchi, Giuseppe Rocca, Laura Gornati, Maria Antonietta Rizzuto, Lucia Salvioni, Ivan Zanoni, Alessandro Gori, Davide Prosperi, Francesca Granucci

**Affiliations:** 1 Department of Biotechnology and Biosciences, University of Milano - Bicocca, Piazza della Scienza 2, 20126 Milan, Italy; 2 Humabs BioMed, Bellinzona, Canton Ticino, Switzerland; 3 Harvard Medical School and Division of Immunology, Division of Gastroenterology, Boston Children’s Hospital, Boston, MA 02115, USA; 4 Istituto di Scienze e Tecnologie Chimiche, National Research Council of Italy (SCITEC-CNR), Via Mario Bianco, 9, 20131 Milan, Italy

**Keywords:** Health sciences, Drugs, Immunology, Immune response

## Abstract

Calcineurin (CN) inhibitors currently used to avoid transplant rejection block the activation of adaptive immune responses but also prevent the development of tolerance toward the graft, by directly inhibiting T cells. CN, through the transcription factors of the NFAT family, plays an important role also in the differentiation dendritic cells (DCs), the main cells responsible for the activation of T lymphocytes. Therefore, we hypothesized that the inhibition of CN only in DCs and not in T cells could be sufficient to prevent T cell responses, while allowing for the development of tolerance. Here, we show that inhibition of CN/NFAT pathway in innate myeloid cells, using a new nanoconjugate capable of selectively targeting phagocytes *in vivo*, protects against graft rejection and induces a longer graft acceptance compared to common CN inhibitors. We propose a new generation of nanoparticles-based selective immune suppressive agents for a better control of transplant acceptance.

## Highlights

• Calcineurin/NFATc2 pathway is required to enable DC migration to draining lymph nodes
• Calcineurin/NFATc2 pathway in DCs is required for type I immune responses activation
• Superparamagnetic iron oxide NPs can be used to efficiently target phagocytes *in vivo*
• Specific delivery of calcineurin inhibitor by NPs to phagocytes induce graft acceptance

## Graphical abstract

**Figure:**
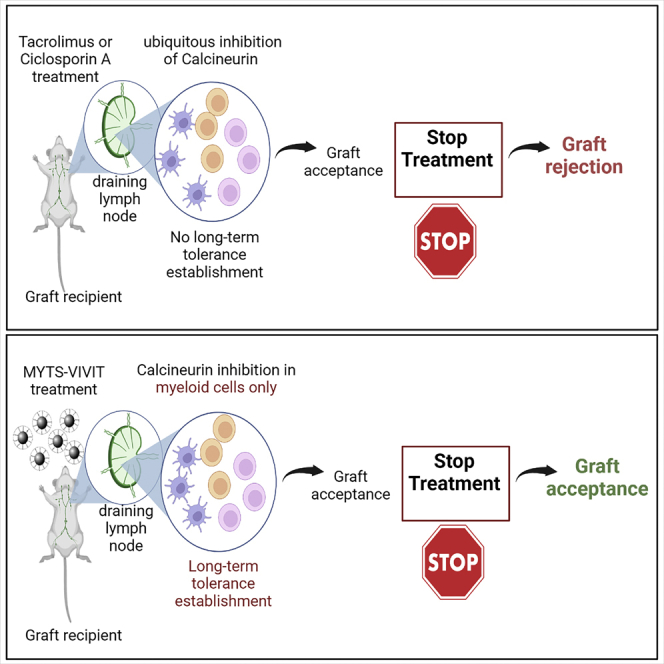

## Introduction

Solid organ transplantation is a viable therapeutic approach that prolongs the life of patients with failing organs. Despite improvements in short-term post-transplant outcomes, the success of the graft is limited by the occurrence of rejection in the presence of antigen mismatches between donor and recipient.

Gold standard therapies to avoid transplant rejection in grafted patients consist of long-life administration of inhibitors of the phosphatase calcineurin (CN), such as Cyclosporin A (CsA) and Tacrolimus (FK506). CN, once activated by a calcium rise in the cells, dephosphorylates the nuclear factor of activated T-cell (NFAT) transcription factor family which migrates to the nucleus and regulates the production of cytokines, like interleukin (IL)-2, which in turn induce antigen-specific T cell proliferation. CsA and FK506 are used to inhibit CN and, in principle, to inhibit IL-2 production by T cells. Nevertheless, we and others have found that CN/NFAT pathway plays a major role, not only in adaptive immunity, but also in the regulation of inflammatory properties of innate immune cells (Zanoni et al., 2009) (Zanoni et al., 2012) (Marongiu et al., 2021) (Herbst et al., 2015) (Goodridge et al. 2007) (Bendickova et al. 2017) (Saito et al., 2022), therefore, its effect may not be limited to T cells but may be extended also to cells of the innate immune system, including dendritic cells (DCs) and macrophages. Although CsA and FK506 are highly efficient immunosuppressors, their use has important limitations as they interfere with the possibility that T lymphocytes become tolerant to the graft, and therefore must be administered for life. Moreover, CsA and FK506 have severe side effects that likely result from the general inhibition of the enzymatic activity of CN, which plays many other physiological roles besides the regulation of IL-2 production in T cells (Kiani et al. 2000). Because DCs are required for T cell activation, inhibiting CN solely in DCs could avoid antigen-specific T cell activation without blocking the induction of T cell tolerance versus the graft. Therefore, we hypothesize that the inhibition of CN in DCs can prevent T cell activation and the consequent graft loss, allowing at the same time the possibility of T cell tolerance induction.

CN interacts with its substrates mainly through the conserved CN binding region that contains the PxIxIT motif (H. Li, Rao, and Hogan 2004) (Y. Li et al., 2020). Blocking the binding of the PxIxIT motif to CN, selectively inhibits substrates that interact with CN through this motif without altering CN phosphatase activity and its ability to interact with other substrates. An optimized PxIxIT peptide, the VIVIT peptide, which has an affinity for CN 50 times higher than the NFAT PxIxIT motif, has been identified by screening a randomized peptide library. This peptide competes for the binding site of CN and inhibits the interaction between CN and the substrates that contain this motif including NFAT (Aramburu et al., 1999). Therefore, using peptides containing the VIVIT sequence represents an interesting alternative to the use of CsA and FK506 as immunosuppressors.

For the delivering of the VIVIT peptide to myeloid cells *in vivo*, we reasoned that colloidal nanoparticles (NPs) could be a valid option for two main reasons: (1) conjugation of peptide with NPs protects peptide from degradation *in vivo* (Spicer et al., 2018) (Jeong et al., 2018); (2) phagocytic cells are a preferential passive target of NPs because they can efficiently phagocytose particulate substances.

Optimal physicochemical characteristics are necessary for NPs to target DCs and/or macrophages. Phagocytosis of nanoparticles by phagocytic cells depends on their surface charge, size, geometry and deformability (Swartzwelter, 2021).

Here we used a new multifunctional NP functionalized with a VIVIT 16mer oligopeptide and with polyethylene glycol (PEG). We found that VIVIT-NPs are efficiently taken up by DCs and enter the cells mainly by endocytosis. Subsequently, NPs are released from the endosome and reach the cytosol, where they efficiently inhibit NFAT nuclear translocation. When administered *in vivo*, graft rejection is prevented even after the interruption of the treatment, as shown using a model of skin transplant. Functionality of non-alloreactive T cells is preserved.

## Results

### NFAT signaling pathway in DCs is necessary to induce efficient T cell activation and graft rejection

The NFAT family encompasses 5 members: NFATc1, c2, c3 and c4 dependent on calcium and CN signaling, and NFATc5 triggered by osmotic stress (Rao et al. 1997). Among the NFAT members, NFATc2 is the most expressed in DCs, hence, initially, we focused our attention on this member (Santus et al., 2017) (Zanoni et al., 2009) (Zanoni et al., 2012) and we took advantage of a mouse model that lacks NFATc2.

**Figure 1:**
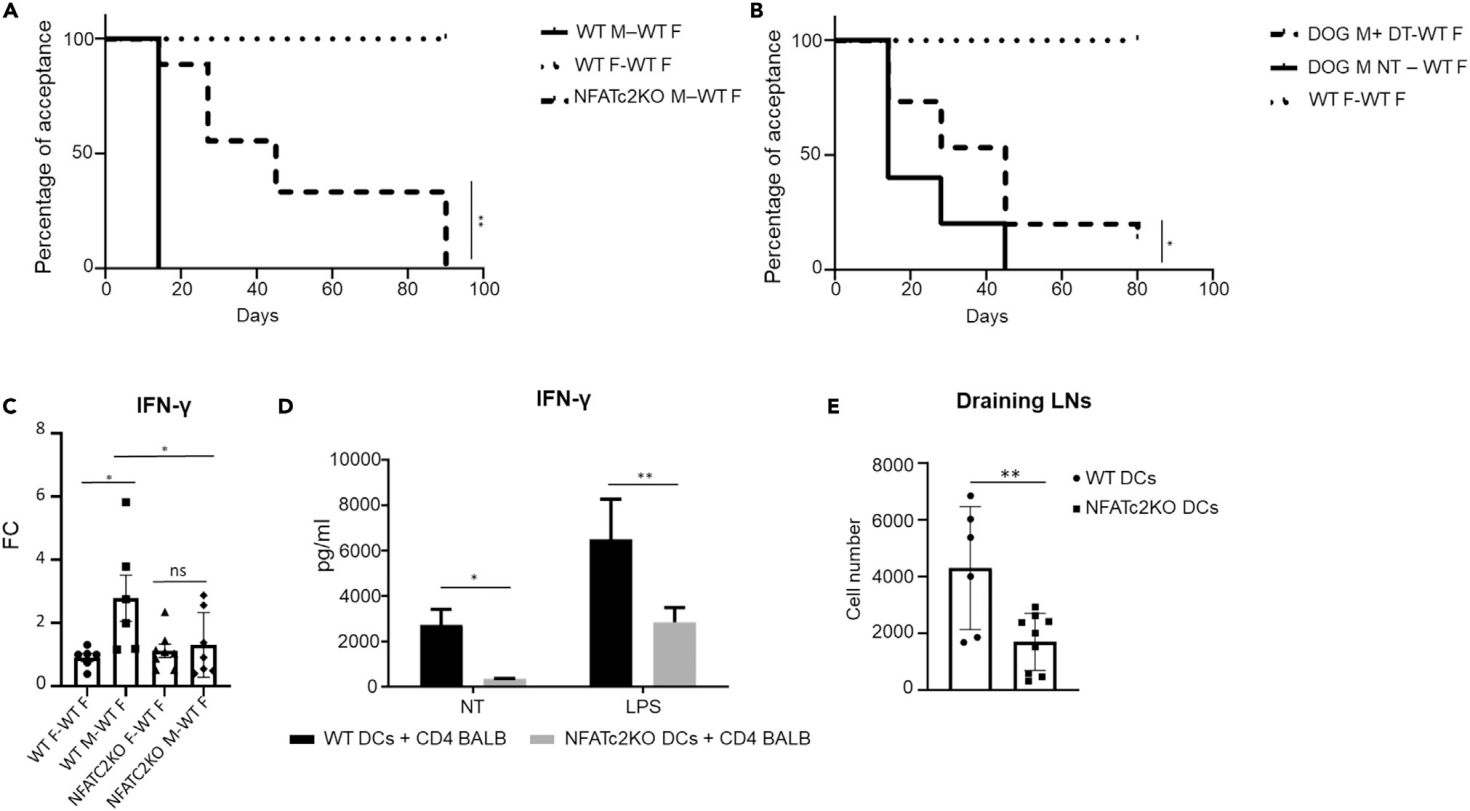
The activation of NFAT signaling pathway in DCs induce efficient T cell activation and graft rejection (A) Kaplan-Meier curve showing the percentage of WT recipients mice undergoing graft acceptance. WT females were transplanted with the skin of donor WT females or WT or NFATC2-deficient males; graft acceptance and rejection were monitored at the indicated time points until day 90 after transplantations. Long-rank (Mantel–Cox) statistical analysis was performed. ∗∗p < 0.001. (B) Kaplan-Meier curve showing the percentage of WT recipients undergoing graft acceptance. Recipient WT females were transplanted with DC-deficient or sufficient male skin or with female skin as control; graft acceptance and rejection were monitored at the indicated time points until day 80 after transplant. Long-rank (Mantel–Cox) statistical analysis was performed, ∗p < 0.05. (C) IFN-γ expression in skin grafts was measured 3 days after transplantation by qPCR. Fold changes (FC) in male over female skin grafts are shown both for WT and NFATc2-deficient skins. Values represent the mean +SE from 6 mice for each group. Statistical significance was determined with one-way analysis of variance, followed by Tukey’s multiple comparisons test, ∗p< 0.05, ns not statistically significant. (D) IFN-γ production by alloreactive T cells stimulated with WT or NFATc2-deficient DCs after 5 days of coculture. DCs were activated or not with LPS (1 μg/mL). Values represent the mean + SD of three independent experiments. Statistical significance was determined with one-way analysis of variance, followed by Tukey’s multiple comparisons test, ∗p< 0.05, ∗∗p< 0.001. (E) Efficiency of migration to draining lymph nodes of NFATC2-deficient and WT DCs measured by FITC painting. Values represent the absolute number of CD11c^+^MHCII^+^ FITC^+^ DCs from three independent experiments. Statistical significance was determined with student’s *t* test, ∗∗p< 0.001.

We used a well-established model of skin graft rejection based on the mismatch of minor histocompatibility antigens (Fernandes et al., 2011) (Kwun et al., 2011). The skin of wild type (WT) or NFATc2-deficient male mice was transplanted onto the dorsum of WT female recipients and followed over time. NFATc2-deficient skin was rejected with a significantly delayed kinetic compared to WT skin (Figure 1A). The rejection process of NFATc2-deficient skin started with a two-week delay compared to WT skin and reached the 100% of graft loss only 80 days after transplantation, whereas all control animals had already lost the graft by day 30. Transplant of skin from female donors into female recipients was used as a control and as expected was well tolerated (Figure 1A). Donor DCs play a major role in graft rejection because they migrate from the graft to the draining lymph node and activate recipient alloreactive T cells through direct alloreactivity. We, thus, used DOG mice, animals that express the simian diphteria toxin (DT) receptor (10^5^-fold more sensitive to DT if compared to its murine counterpart) predominantly in DCs under the control of CD11c (DC specific marker) promoter (Hochweller et al., 2008). DOG mice were deprived of DCs by administration of DT before skin removal for transplantation (Figure S1 for efficiency of DC deprivation) and the kinetic of rejection followed over time. As expected, DC deprivation caused a delay in graft rejection (Figure 1B). The delay observed when DCs were depleted in the donor skin was similar to the delay observed when NFATc2-deficient skin was transplanted. This observation supported our hypothesis that NFATc2 might play a role in controlling the ability of DCs to activate T cells.

Because activated alloreactive T cells responsible for graft rejection (CD8^+^ or Th1 lymphocytes) produce IFN-γ (Slavcev et al., 2015) (Bhat et al., 2017), we measured the local production of IFN-γ in the transplanted WT or NFATc2-deficient tissues. We found that the amounts of IFN-γ transcripts in grafts derived from WT males transplanted in WT female was elevated when compared to the level exhibited by the skin from WT females transplanted into WT female recipients (Figure 1C). In addition, NFATc2-deficient male skin grafted in WT female recipients displayed an IFN-γ transcription comparable to the one of the accepted transplants (Figure 1C), suggesting that the absence of NFATc2 in the graft reduces the production of one of the fundamental mediators of rejection. Moreover, mixed lymphocyte reaction (MLR) assay was performed to evaluate a possible role of NFATc2 in DCs to activate alloreactive T cells. Co-culture of T cells from BALB/c mice and bone marrow derived DCs (BMDCs) from NFATc2-dieficient C576BL/6 mice was performed. Once plated, T cell activation was evaluated after 5 days of culture, by measuring IFN-γ in the supernatants. IFN-γ production was significantly lower in co-cultures of T cells and NFATc2-deficient BMDCs compared to the controls (Figure 1D). NFATc2-deficient BMDCs did not differ from WT BMDCs in terms of activation markers expression both in resting conditions and after LPS exposure (Figure S2). Altogether these data suggest that the lack of NFATc2 in DCs causes a decrease in their capacity to activate Th1 T cell responses.

Under inflammatory conditions, tissue resident DCs migrate to the T cell area of lymph nodes draining the inflamed region to eventually activate T cells. Therefore, to better define the role of NFATc2 in DCs *in vivo*, we also investigated the migratory capacity of WT and NFATc2-deficient DCs using the FITC painting method (Platt et al., 2013). We found that the efficiency with which NFATc2-deficient DCs reached draining lymph nodes was severely affected compared to WT DCs (Figure 1E).

Therefore, the absence of NFATc2 in DCs and, consequently, of its transcriptional activities, reduced DC ability to elicit IFN-γ production by T cells and reduced the migration of DCs from the inflamed tissue into the draining lymph node.

In the context of organ transplantation, the lack of NFATc2 determines a significant delay in the rejection of mismatched transplants, suggesting that NFAT signaling pathway in DCs is critical for the instauration of alloreactivity.

### A novel tool to inhibit the NFAT pathway in innate immune cells

The absence of NFATc2 in the transplant was sufficient to delay the rejection process possibly because of the reduced ability of NFATc2-deficient DCs to migrate to the draining lymph nodes and to stimulate the production of IFN-γ by T lymphocytes. Our prediction was that inhibition of all NFATc members in antigen presenting cells (APCs) could further reduce DC function, thereby further improving graft survival. Because our aim was to inhibit NFATc in innate immune myeloid cells and, specifically, in DCs, we required a tool to inhibit all NFAT members in these cells. We decided to use the VIVIT peptide that efficiently blocks the interaction of NFAT with CN. We also reasoned that NPs could be a good option, both to protect the VIVIT peptide from degradation *in vivo* and to target phagocytic cells.

Colloidal NPs useful for this study were designed following a few basic criteria, including high colloidal stability in aqueous media, compatibility with biological environment and tunable surface charge. Compared to other kinds of colloidal NPs, including, for instance, broadly utilized gold, silica and polystyrene (Ahmad, 2017) (S. Liu et al., 2019) (Frick et al., 2012), superparamagnetic iron oxide NPs (IONPs) seem to be less active in inducing antigen cross-presentation and migration of DCs (deVries et al., 2005). In addition, cationic NPs induce much higher effect on DCs in comparison to anionic NPs (Mou et al., 2017) (Jia et al., 2018). On this basis, we reasoned that small IONPs coated with negatively charged amphiphilic polymer suitable for further functionalization could be the best candidate for our study. The NP core consisted of an iron oxide nanocrystal tightly wrapped by a polyanionic amphiphilic polymer that provided excellent colloidal stability in aqueous solution, and multiple functional groups suitable for straightforward bioconjugation (Truffi et al., 2018). We refer to these nanoparticles as MYTS-VIVIT (Figure S3A) (Marongiu et al., 2021).

We also explored the possibility to develop VIVIT nanoconjugates, in which the same amphiphilic polymer, utilized for IONP coating in MYTS, was exploited to form stable micelles in the absence of an iron core (Figure S3B). In addition, VIVIT NPs obtained by VIVIT incorporation in a well-established bionanoparticle, which has already proven to be able to escape the endosomes after cellular uptake, namely the H-Ferritin (HFn) (Rizzuto et al., 2021) (He et al. 2019), were also synthesized and assessed for comparison (Figure S3C). Representative TEM images of MYTS-VIVIT, PMDA-VIVIT and HFn-VIVIT are shown in Figures S3A–S3C.

MYTS-VIVIT were synthesized in two steps according to our established protocol for the preparation of multifunctional superparamagnetic NPs (Mazzucchelli et al., 2013b) (Fiandra et al., 2013). First, iron oxide nanocrystals (11.7 ± 1.4 nm as determined by transmission electron microscopy, TEM, Figure S3D) stabilized by a long-chain surfactant (e.g., oleic acid or oleylamine) were coated with an amphiphilic polymer termed PMDA (poly[isobutylene-*alt*-maleic anhydride]-graft-dodecylamine, MW ∼11.5 kDa) (Figure S4A) obtained by condensation of poly[isobutylene-*alt*-maleic anhydride] (MW ∼6 kDa) and dodecylamine (Lin et al., 2008) (75% of available maleic groups on the polymer backbone) followed by basic hydrolysis, resulting in highly colloidally stable dispersion of NPs that we referred to as MYTS (Truffi et al., 2018) (Mazzucchelli et al., 2013a) (Figure S4B). MYTS were characterized by dynamic light scattering (DLS) and exhibited hydrodynamic size of 23 nm with uniform size distribution in aqueous solution. ζ potential analysis confirmed a characteristic strong negative surface charge of −50 mV (Table 1). The VIVIT conjugation strategy was designed exploiting a disulfide bridge-bearing linker between the peptide and the NP, which allowed the nanoconjugate to trigger the release of the peptide on contact with the reducing cytosolic environment. To do so, the carboxyl groups of the coating polymer were first modified with ethylenedioxy bis(ethylamine) (EDBE) and further functionalized with a sulfhydryl-reactive crosslinker (succinimidyl 3-[2-pyridyldithio]propionate, SPDP) (Marongiu et al., 2021) (Figure S4B). Quantification of VIVIT loading was performed using a dye-labeled (rhodamine isothiocyanate) peptide, resulting in 17 VIVIT molecules per NP on average. The same disulfide bridge strategy was adopted to immobilize PEG molecules (5 kDa) on the outer polymer to improve the long-term NP circulation *in vivo*.

**Table 1:** Chemical physical characterization of nanoparticles and nanoconjugates used in this work

Sample	DLS (nm)	std (nm)	Zeta potential (mV)	std (mV)	Loading (n. VIVIT/NP)
MYTS	22.9	1.4	−49.8	1.5	–
MYTS-VIVIT	22.6	2.4	−45.1	1.5	17.0
MYTS-PEG	26.2	1.6	−49.1	1.8	–
PMDA	5.4	0.2	−40.1	4.5	–
PMDA-VIVIT	5.5	0.2	−29.5	4.2	0.6–1.6^a^
PMDA-PEG	5.5	0.6	−30.9	3.9	–
HFn	13.0	3.1	−11.6	5.6	–
HFn(VIVIT)	13.4	3.0	−10.2	4.4	38.4

All measurements were conducted in 10 mM ionic strength, pH 7.35.

a The value is size-dependent (in the range 5–9 nm) calculated at a density of 1 mg/mL.

Stable PMDA micelle dispersion in aqueous solution was prepared following the procedure described in a previous study (Rotem et al., 2020). In that work, PMDA were reported to form micelles in the range 5–12 nm in aqueous solution, although size and stability were strictly dependent on the experimental pH and ionic strength. For instance, under physiologic conditions, PMDA micelles were 10.6 ± 0.8 nm in size with a strong negative surface charge of −40 ± 5 mV. Importantly, PMDA micelles proved to be able to penetrate the plasma membrane and diffuse into the cytoplasm (Rotem et al., 2020). Thus, PMDA may represent an ideal candidate polymer coating to enhance the NP entry into the cytoplasm. Under the conditions used in the present study (10 mM ionic strength, pH 7.35), TEM analysis displayed uniform spherical micelles in the range 16 ± 4 nm with quite narrow size distribution (Figure S4C). However, the hydrodynamic size of PMDA was 5.5 nm (number distribution) with a zeta potential of −40 mV (Table 1). In this case, a difference from TEM analysis was expected because of the soft nature of the material, coming from the drying process associated with grid preparation. For this reason, we estimated that DLS was presumably a better indication of the real size of PMDA micelles and of their nanoconjugates in solution. Micelles were easily modified with EDBE and functionalized with SPDP to allow the immobilization of VIVIT and PEG molecules according to the same strategy used for MYTS (Figure S4C).

A third model NP system for VIVIT delivery was HFn. VIVIT loading of HFn was achieved exploiting the disassembly and reassembly reaction, adjusting our previous protocol (Rizzuto et al., 2021) (Bellini et al., 2014). This protocol is usually utilized for incorporation of small molecules (Bellini et al., 2014) (Rizzuto et al., 2021) (Mazzucchelli et al., 2017). Here we optimized the reaction conditions to achieve the best loading efficiency with a biomolecule such as the VIVIT peptide. After HFn dissociation at acidic pH, a 90-fold molar VIVIT excess was added to the solution and pH was gradually restored to 7.4 allowing HFn cages to slowly reassemble encapsulating a certain amount of VIVIT (FigureS4D). DLS analysis of HFn(VIVIT) revealed that, as expected, NPs overall dimension was not significantly changed after the loading reaction, showing a hydrodynamic size of 13.4 ± 3.0 nm as compared to 13.0 ± 3.1 nm for HFn alone, with quite uniform size distribution in aqueous solution. Also, the zeta potential did not change significantly, confirming that VIVIT molecules were incorporated inside the inner cavity and not adsorbed onto the protein surface.

VIVIT loading in MYTS-VIVIT, PMDA-VIVIT and HFn(VIVIT) was determined by fluorescent measurements using dye-labeled VIVIT, providing on average 17, 1, and 38 VIVIT molecules per NP, respectively. DLS, zeta potential and loading data for all synthesized nanoconjugates are summarized in Table 1.

### Inhibition of NFAT translocation by VIVIT-NPs and uptake of NPs by phagocytic cells

**Figure 2:**
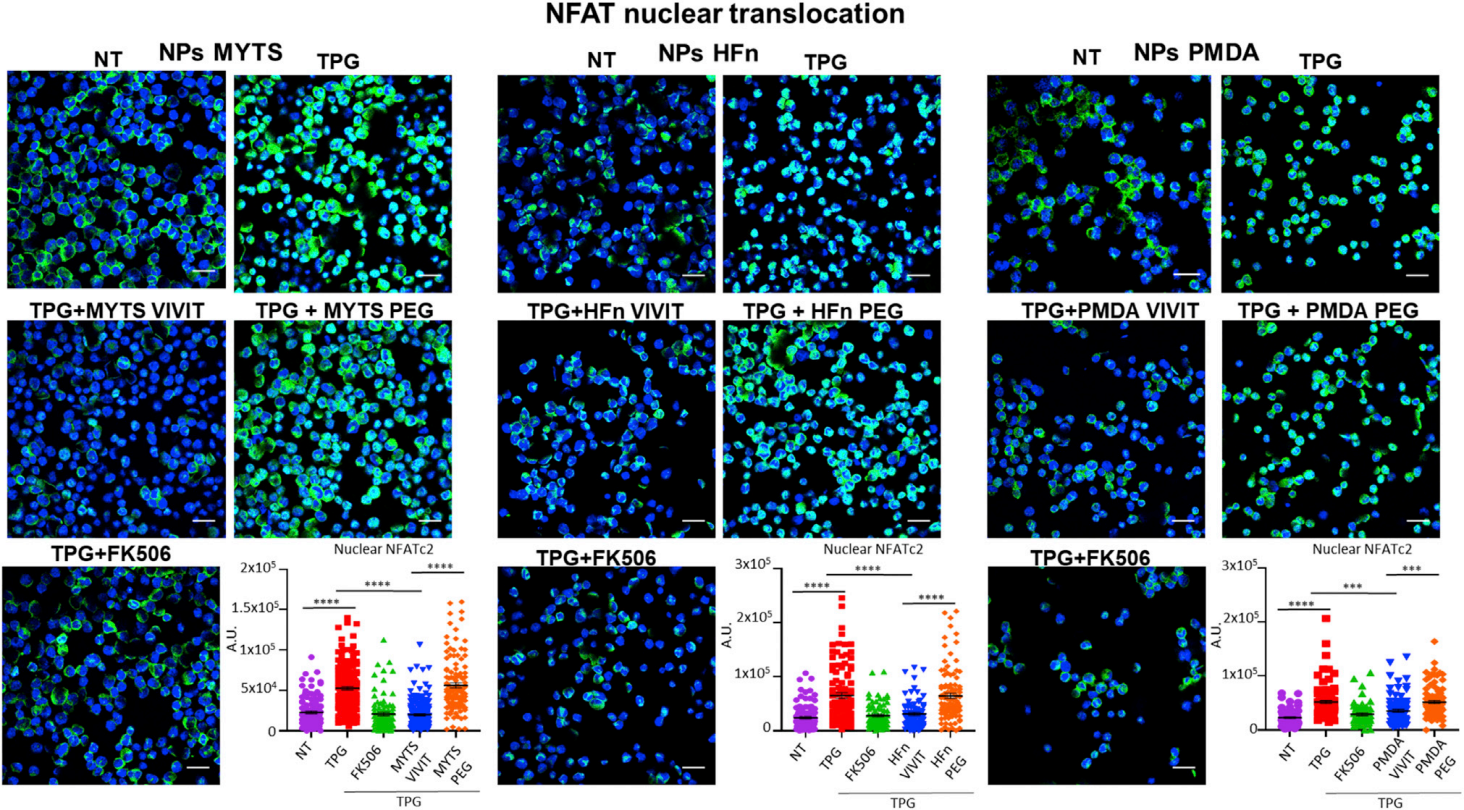
VIVIT nanoparticles inhibit the nuclear translocation of NFAT A20 cells expressing NFAT4-GFP fusion protein were pretreated with the indicated NPs (50 μg/mL) or with FK506 (10 ng/mL) and then stimulated with thapsigargin (TPG, 50nM) for 40 min. Nuclear translocation of NFAT4-GFP fusion protein was evaluated by confocal microscopy. Representative confocal images for each condition are shown (nuclei are marked in blue, scale bar = 25um) as well as the quantification of the NFAT4-GFP nuclear signal. Quantification data are presented as mean+SD. Statistical analysis was performed with one-way analysis of variance followed by Tukey’s multiple comparisons test, ∗∗∗∗p< 0.0001, ∗∗∗p< 0.001 *n* = 100 cells.

The three different types of NPs were first analyzed for their capacity to inhibit NFAT activation. A20 myeloma cells were transfected with a vector expressing NFATc4-GFP fusion protein and nuclear NFAT translocation was induced by the exposure to thapsigargin in the presence or not of NPs. All VIVIT-conjugated NPs affected nuclear translocation of NFAT with similar efficiency (Figure 2).

**Figure 3:**
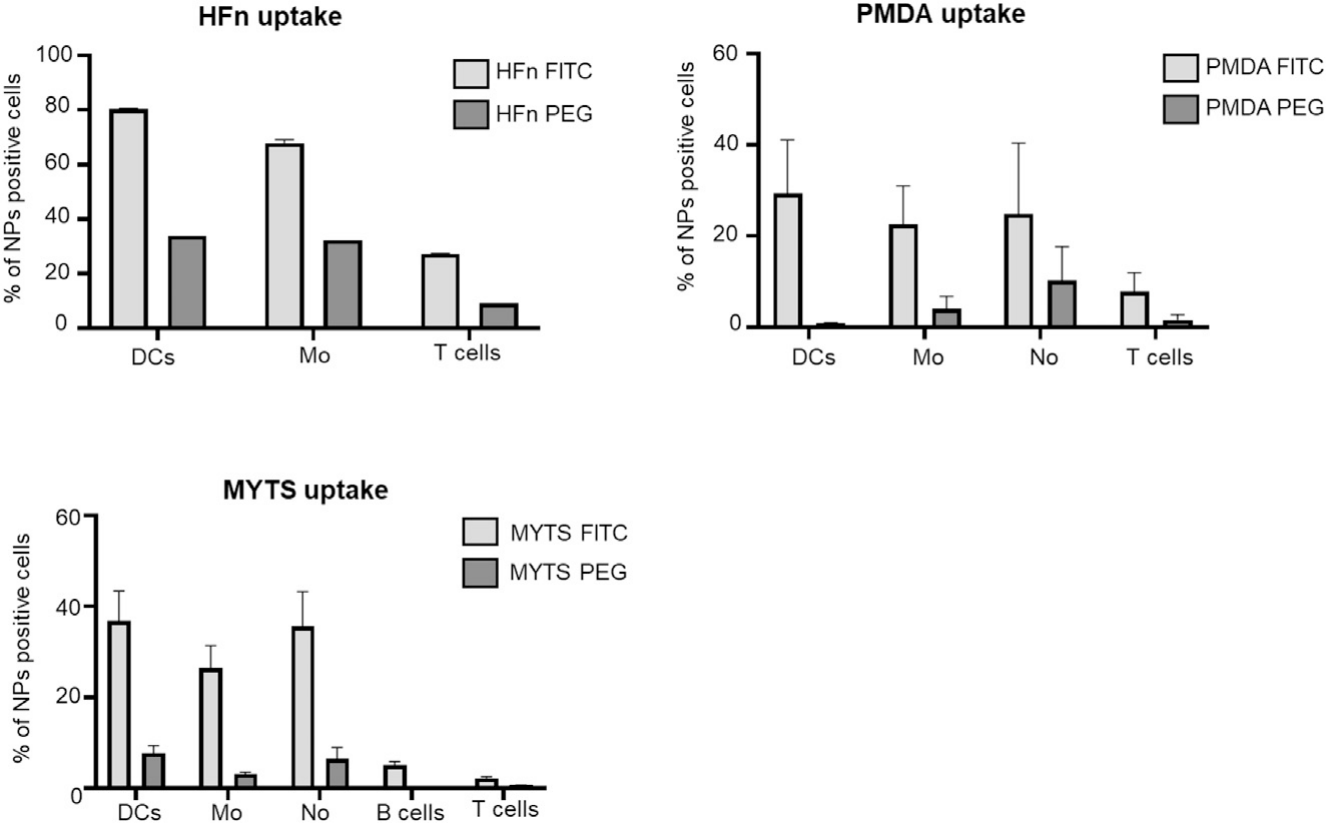
MYTS NPs are preferentially taken up by phagocytes *in vivo* The distribution of MYTS, HFn or PMDA NPs, 90 min after i.p. administration (100μg/mouse), was analyzed in the spleens of treated mice. The percentage of NP-positive DCs (CD11c^+^MHCII^+^ cells), macrophages (CD11b^+^ cells), neutrophils (Ly6G^+^CD11b^+^ cells), T (CD3^+^ cell) and B (CD19^+^ cells) lymphocytes are shown for each type of NP. Data are presented as mean +SE from three different mice.

Because we aimed at inhibiting, *in vivo*, NFAT activation in phagocytic cells but not in T cells, we investigated the uptake of the three types of NPs *in vivo*. We injected fluorescently labeled MYTS, PMDA micelles, and HFn, and we measured the uptake by DCs, macrophages, neutrophils, T and B cells. HFn and PMDA were efficiently taken up by phagocytic cells but also, although to a lower extent, by T cells; diversely, MYTS NPs were taken up predominantly by phagocytic cells and only negligibly by T and B cells (Figure 3) (Marongiu et al., 2021). Therefore, taking advantage of the higher selectivity toward target cells, we decided to use MYTS-VIVIT for further *in vitro* characterizations and *in vivo* studies.

### MYTS NPs entry and endosomal escape in DCs

As already mentioned, the VIVIT peptide interferes with the interaction between CN and NFAT occurring in the cytoplasm. Hence, a critical issue for an effective VIVIT delivery is the propensity of the nanocarrier to penetrate the cell membrane and diffuse into the cytosol to release the peptide therein. Most colloidal NPs are known to enter the host cell by endosomal routes which makes the endosomal escape a crucial step of intracellular drug delivery. This is particularly relevant for phagocytic cells, which engulf NP clusters and aggregates inside large vesicular endosomes that develop into late endosomes and eventually fuse with lysosomes to favor digestion and elimination of the NP cargo (Rennick et al. 2021).

**Figure 4:**
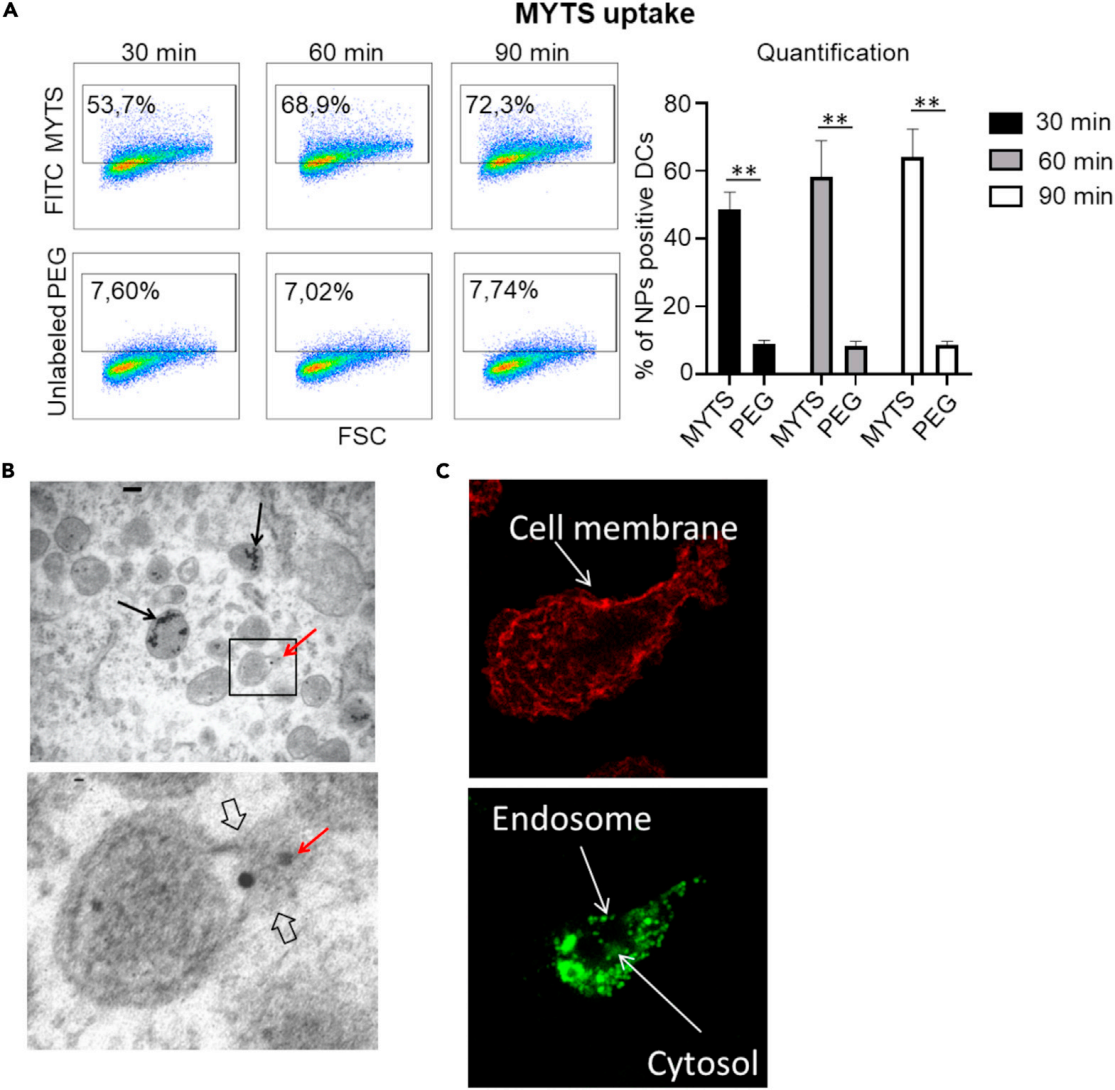
MYTS NPs can escape the endosomal compartment (A) BMDCs were incubated for the indicated time points with 50 μg/mL of MYTS-FITC. Cells were then analyzed with flow cytometry for the internalization of NPs. DCs were identified as CD11c^+^MHCII^+^ cells. Data shown in the quantification graph are the mean +SD from three independent experiments. Statistical analysis was performed with one-way analysis of variance followed by Tukey’s multiple comparisons test, ∗∗p< 0.001. (B) TEM analysis of BMDCs treated with 50 μg/mL of MYTS for 90 min. Black arrows indicate NPs confined in endosomes; red arrows indicate NPs present in the cytoplasm. Panel insets represent higher magnification of the selected area. (C) Representative confocal microscopy images showing the intracellular distribution of MYTS-FITC in BMDCs after 5 h of co-culture; cell membrane is shown in red.

DCs were treated with fluorescently labeled MYTS in a set of experiments aimed at determining their effectiveness in escaping the endolysosomal route. First, MYTS entry in DCs was investigated by FACS analysis. DCs were incubated with labeled-MYTS NPs and the percentage of fluorescent DCs evaluated. As shown in Figure 4A, DCs *in vitro* were able to uptake the NPs with high efficiency.

Next, cells exposed to NPs were isolated and observed at TEM for direct ultrastructural imaging. In Figure 4B, MYTS were detected partly confined in endosomes (black arrows), partly dispersed in the cytoplasm (red arrows) and also snapshotted in the attempt to evading the endosomal membrane (Figure 4B; more pictures in the Figure S5). Confocal microscopy using dye labeled MYTS confirmed the capacity of the MYTS NPs to evade the endosomal confinement. Indeed, recent studies suggest that high local accumulation of dye inside the early and late endosomes combined with progressive lowering of pH partially quenches the fluorescence intensity, leading to a punctate signal distribution within the cell. In contrast, after endosomal escape, the NPs disperse into the cytosol, resulting in intense and diffuse fluorescence (Smith et al., 2019). Figure 4C shows MYTS distribution in the endosome and cytosol 5h after uptake. Intense fluorescent spots attributable to NP confinement into early endosomes (Figure 4C) and diffuse green fluorescence widespread in the cell cytosol were clearly visible confirming that endosomal escape occurred under our experimental conditions (Figure 4C).

### *In vivo* treatment with MYTS-VIVIT does not affect T cell activation

**Figure 5:**
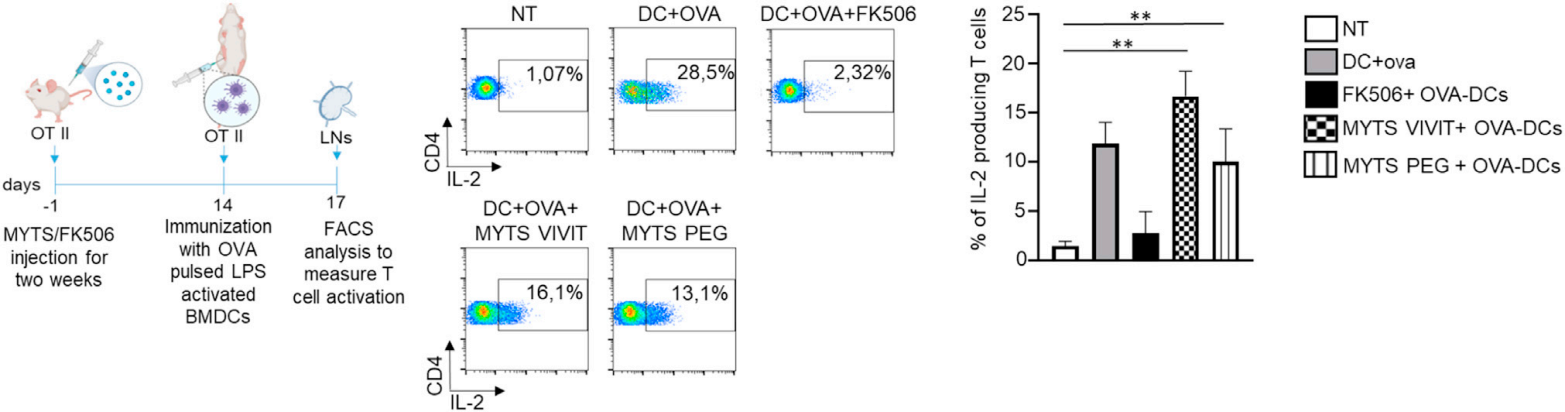
*In vivo* treatment with MYTS-VIVIT does not affect the capacity of T cells to respond to immunization (Upper panel) Scheme of the experiment. OT II mice were treated with MYTS-VIVIT or MYTS-PEG every other day or with FK506 every day for two weeks. After the two-week treatment, OVA-pulsed and LPS activated BMDCs were injected in the footpad of treated mice and draining lymph nodes collected after 72 h to assess activation of CD4^+^ OVA-specific T cell by means of IL-2 production. (Lower panel) IL-2 production by OVA-specific T cells from mice that received the indicated treatments. Data are shown as mean +SD and represent of two independent experiments 4 animals per group in total. Statistical analysis was performed with one-way analysis of variance followed by Tukey’s multiple comparisons test, ∗∗p< 0.001.

Next, we tested the capacity of MYTS-VIVIT to affect T cell responses *in vivo*. To confirm that T cells were unaffected upon the administration *in vivo* of MYTS-VIVIT, we administered to OT II mice that express a T cell antigen receptor (TCR) specific for a peptide of ovalbumin (OVA), MYTS-VIVIT or MYTS-PEG or FK-506 every other day for 2 weeks. We, then, immunized these OT II mice with OVA-pulsed LPS-activated BMDCs in the footpad, and we analyzed T cell activation in draining lymph nodes, 72 h after DC injection. As shown in Figure 5, mice who received FK-506 did not mount an adaptive response, as indicated by the inability of T cells to produce IL-2. Conversely, MYTS-VIVIT treatment did not alter the capacity of T cells to undergo activation, as shown by their ability to produce IL-2 (Figure 5). These data indicate that T cells are not affected by the treatment with VIVIT-conjugated NPs because they do not incorporate the NPs, whereas, as expected, they are affected by FK-506. Taken together, these data highlight the potential of MYTS-VIVIT as a novel tool to preferably inhibit NFAT activation in phagocytes, without affecting T cells, which therefore may retain the ability to be tolerized.

### MYTS-VIVIT treatment confers long-term protection upon skin transplant

**Figure 6:**
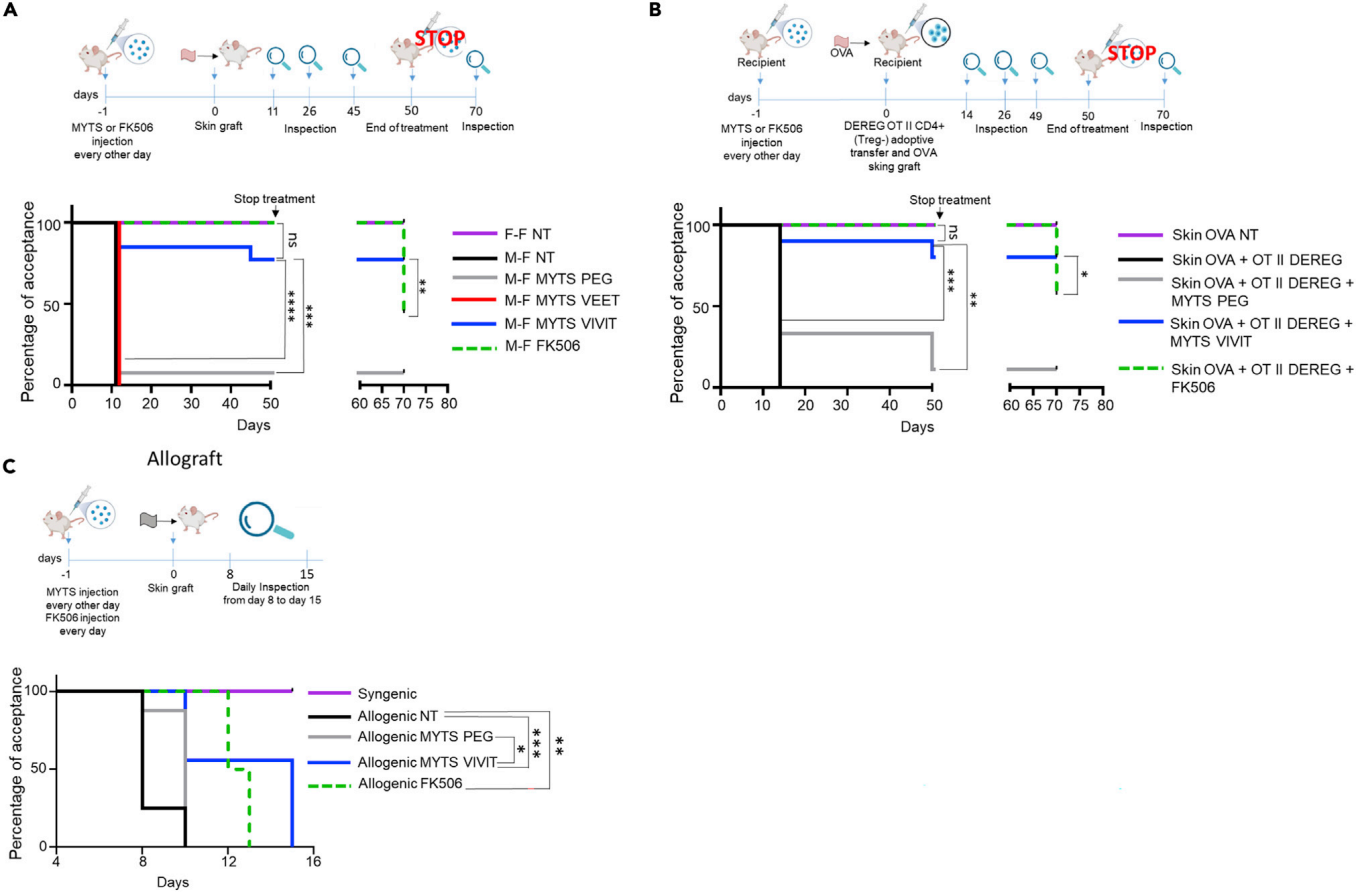
MYTS-VIVIT treatment induce long-term skin transplant acceptance (A) Upper panel, scheme of the experiment. Recipient mice were transplanted at day 0 and treated with NPs or FK506 every other day until day 50 after transplantation starting from day −1. Observations were performed at the indicated time points after surgery. Treatments were stopped at day 50 and mice observed again 20 days later (day 70). Lower panel, Kaplan-Meier curve showing the percentage of recipient mice undergoing graft acceptance. The arrow indicates the end of the treatment. Long-rank (Mantel–Cox) statistical analysis was performed, ∗∗∗p < 0.001, ∗∗∗∗p < 0.0001. (B) Upper panel, scheme of the experiment. Recipient mice were adoptively transferred at day 0 with OT II cells deprived of Treg cells and then transplanted with skin samples from K5mOVA mice. Transplanted recipients were treated with NPs or FK506 every other day starting from day −1 until day 50 after transplantation. Observation was performed at the indicated time points after surgery. Treatments were stopped at day 50 and mice observed again 20 days later (day 70). Lower panel, Kaplan-Meier curve showing the percentage of recipient mice undergoing K5-mOVA skin graft acceptance. The arrow indicates the end of the treatment. Long-rank (Mantel–Cox) statistical analysis was performed, ∗∗∗p < 0.001. (C) Upper panel, scheme of the experiment. Recipient mice were transplanted at day 0 with allogeneic skin and treated with NPs every other day or FK506 every day after transplantation starting from day −1. Inspections were performed daily starting from day 8 after transplantation. Lower panel, Kaplan-Meier curve showing the percentage of recipient mice undergoing graft acceptance. Long-rank (Mantel–Cox) statistical analysis was performed, ∗p < 0.05; ∗∗p < 0.01; ∗∗∗p < 0.001.

To investigate whether the MYTS-VIVIT was able to interfere with graft rejection, we performed skin transplant in two different settings: (1) male in female grafts; (2) recipients with high frequency of T cells specific for an antigen expressed by the transplanted skin. In the first setting, the following groups were analyzed: the control group (female-derived skin into female recipients); the rejection group (male-derived skin into female recipients); male-derived skin into female recipients treated with MYTS-VIVIT or MYTS-VEET (control) or MYTS-PEG (control) or FK-506 every other day until the end of the experiment. Mouse inspection was performed starting from day 11 after surgery. First, to test the activity of NPs *in vivo* we used a transplant model with limited antigen mismatch because skin grafts are the most strongly rejected. As reported in Figure 6A, by the first day of inspection, most or all control animals (untreated mice or animals treated with MYTS-VEET or MYTS-PEG) who received male skin had rejected the graft. In contrast, most of the MYTS-VIVIT-treated animals accepted the graft for the entire time window observed as did the control group treated with FK-506. Thus, the inhibition of CN in phagocytes confers protection on mismatched transplant.

In the second transplantation model we combined K5-mOVA-derived skin with OT II T cells, infused in recipient animals, to evaluate antigen-specific responses against the graft (Figure 6B). K5-mOVA is a transgenic animal model which expresses a membrane-bound form of OVA (mOVA) under the promoter of keratin 5 (K5) in the epidermal and hair follicular keratinocytes residing in the skin. Furthermore, to avoid biases because of the presence of OT II regulatory T cells (Tregs) in the CD4^+^T cell compartment we took advantage of another transgenic animal model: DEREG OT II mouse. DEREG mice bears the DEREG cassette that allows the expression of the simian DT receptor under the control of Foxp3 promoter (Lahl and Sparwasser, 2011), therefore FoxP3^+^ Treg cells can be eliminated on DT administration to the mice. DEREG mice were backcrossed with OT II transgenic animals for one generation to obtain DEREG x OT II F1 mice. We treated these animals with DT, collected CD4^+^T cells deprived of Treg cells, and adoptively transferred them into female recipient mice on the day of the K5-mOVA-derived skin transplantation. Recipients were then treated with either MYTS-VIVIT or MYTS-PEG and monitored for rejection. Notably, even in this potent antigen-specific setting, mice administered with MYTS-VIVIT did not reject skin graft, whereas MYTS-PEG-treated animals did (Figure 6B).

To evaluate whether the protective effect observed in mice treated with the MYTS-VIVIT is prolonged over time, we interrupted the administration of NPs after 50 days in both transplantation models and evaluated graft rejection after additional 20 days. Surprisingly, MYTS-VIVIT-treated animals did not reject the grafts even in the absence of the treatment, whereas around 50% of FK-506-treated animals underwent rejection (Figures 6A and 6B), significant difference between the two groups were, indeed, observed. This indicated that treatment with MYTS-VIVIT, that inhibit the CN/NFAT signaling pathway exclusively in phagocytic cells and not in T cells, induces long-term skin graft tolerance at least when the mismatch between the donor and the recipient is limited to minor histocompatibility antigens.

Given the high efficiency of MYTS-VIVIT NPs in protecting against rejection when male skin is transplanted into a female, we tested the effectiveness of these NPs compared to FK-506 in protecting against rejection in an allograft setting. Skin from BALB/c animals was transplanted in C57BL/6 mice and rejection evaluated over time starting from day 8 (day of dressing removal). Under these conditions, MYTS-VIVIT NPs were significantly more efficient in protecting against graft rejection than control NPs (Figure 6C), confirming that interfering with CN activity exclusively in myeloid cells without affecting T cells can be sufficient to ensure graft acceptance.

## Discussion

Acute graft rejection is the result of a complex series of events involving both innate and adaptive immunity, in which DCs play a major role by presenting allo-antigens to recipient lymphocytes. NFAT does play a role in determining both the activation and the tolerogenic state of T lymphocytes. The inhibition of the NFAT pathway in T cells efficiently blocks effector T cell generation and graft rejection but interfere also with the induction of T cell tolerance. Therefore, targeting accessory cells required for T cell activation to make them tolerogenic is an alternative approach to control T cell activation without compromising their capacity to become unresponsive to the graft. CN inhibitors have widely been used to inhibit adaptive responses and to prevent alloreactivity without considering the biological effects that these drugs may have in other immune and non-immune cells. FK-506-treated DCs suppress the proliferation of T cells induced by mature DCs, suggesting a potential tolerogenic activity by tacrolimus-treated DCs (Ren et al., 2014). Therefore, the induction of tolerogenic DCs may be part of the benefit of therapy also with CsA and FK506.

Here, we have found that the inhibition of the NFAT signaling pathway in DCs rather than in T cells, after solid organ transplantation, leads to graft acceptance for a long period of time. NFAT pathway abrogation in DCs induces a sort of tolerogenic profile that abrogate Th1 responses (Ren et al., 2014) (Imai et al., 2007) (Lee et al., 2005) (Szabo et al. 2001). Indeed, recipients’ T lymphocytes are unaffected by the NP treatment, hence they can activate and skewing their response, dependent on the status of DCs. There are at least two mechanisms through which NFAT regulates DC functions: (1) it is required for DC migration; (2) it is required to make DCs capable of inducing type I adaptive responses by eliciting IFN-γ production by lymphocytes. Targeting this pathway with nanodrugs proved to be an efficient alternative to commonly used CN inhibitors to avoid graft rejection at least in experimental models.

MYTS NPs are taken up predominantly by phagocytes and, while enter the cells through the endocytic pathway, they efficiently escape the endosome and reach the cytosol, thus representing a valuable delivery system to transfer a cargo inside phagocytic cells. The intrinsic propensity of MYTS NPs to efficiently escape the endosomes and enter the cytoplasm, combined with the ease of immobilization of different kinds of molecules on their surface, may represent a great opportunity for the delivery of various drugs and genes. Interestingly, MYTS-VIVIT was effective, unlike FK-506, in inducing long-term graft survival, even after discontinuation of treatment. This suggests that MYTS-VIVIT can induce a form of tolerance versus the grafts that can be exploited to evaluate new anti-rejection approaches.

After 40 years from their discovery, CN inhibitors like CsA and FK-506 remain the standard of care, at least, in organ transplantation, which is the field most revolutionized with the introduction of these drugs. Nevertheless, CsA and tacrolimus entail several side effects. Indeed, CN inhibitors act upstream NFAT activation by inhibiting the phosphatase activity of CN that modulates other transcription factors, such as NF-κB, AP-1, Elk1 and CREB (Dolmetsch et al. 1998) (Frantz et al., 1994) (Oetjen et al., 2005) (Sugimoto et al. 1997). In addition, CN takes part to other signaling pathways as that triggered by tyrosine kinases downstream of TCR (Dutta et al., 2017), TGF-β or the MAPK cascade (Q. Liu, Busby, and Molkentin 2009) (Ninomiya-Tsuji et al., 1999), hence the abrogation of CN activity via CN inhibitors leads to several side effects besides the inhibition of the NFAT pathway. The adverse events reported during the administration of these drugs regard neurotoxicity, nephrotoxicity, vascular toxicity and remain a major challenge.

The results of this work demonstrate that MYTS-VIVIT represents an extremely simple and powerful tool to inhibit the NFAT signaling pathway *in vivo* in innate immune cells. The large versatility of this tool opens new important possibilities of intervention on the CN-NFAT pathway *in vivo*, well beyond transplant rejection, offering a conceptually innovative arrow in the quiver of the next generation medicine that is expected to improve treatment opportunities against acute and chronic inflammatory diseases.

### Limitations of the study

A limitation of this work is that we used mice as a model system to study the immune response leading to allograft rejection, in conditions in which the NFAT signaling pathway is inhibited in myeloid cells *in vivo*. The response we observed may not fully recapitulate the response that might be found in humans. It could be interesting to investigate the ability of NFAT-inhibited human DCs to activate an alloreactive T cell response in a humanized mouse model. Moreover, we did not optimize the efficiency of peptide delivery to the cytosol of myeloid cells *in vivo* and we did not use combination therapies to improve the efficiency of the nanoparticle treatments *in vivo* in the full alloreactive model. This optimization could lead to more efficient allograft acceptance and development of Tregs that prevent graft rejection.

## STAR★Methods

### Key resources table

**Table:** 

REAGENT or RESOURCE	SOURCE	IDENTIFIER
**Antibodies**		
PE anti-mouse/human CD11b (clone M1/70)	Biolegend	Cat. # 101208
Percp cy5.5 anti-mouse CD3 (clone 17A2)	Biolegend	Cat. # 100218
PE anti-mouse CD4 (Clone GK1.5)	Biolegend	Cat. # 100408
PEcy7 anti-mouse CD11c (clone N418)	Biolegend	Cat. # 117318
APC anti-mouse MHCII (clone AF-120.1)	Biolegend	Cat. # 116418
Pacific blue anti-mouse Ly6G (clone 1A8)	Biolegend	Cat. # 127612
APC/Cyanine7 anti-mouse CD19 (Clone 6D5)	Biolegend	Cat. # 115530
FITC anti-mouse CD86 (clone PO3)	Biolegend	Cat. # 105110
FITC anti-mouse IL-2 (clone JES6-5H4)	Biolegend	Cat. # 503806
LPS from *E*. *coli*, Serotype O55:B5 (TLRGRADE)	Enzo Life Sciences	Cat. # ALX-581-013
Diphtheria Toxin from *Corynebacterium diphtheriae*	Sigma-Aldrich	Cat. # D0564

**Chemicals, peptides, and recombinant proteins**		

FK506 monohydrate	Sigma-Aldrich	Cat. # F4679
Thapsigargin	Sigma-Aldrich	Cat. # T9033
Poly(isobutylene)-alt-maleic anhydride	Merk Life Science S.r.l.	Cat# 531278
Dodecylamine	Merk Life Science S.r.l	Cat# 44172
2,2’-(Ethylenedioxy)bis(ethylamine) (EDBE)	Merk Life Science S.r.l.	Cat# 385506
N-(3-Dimethylaminopropyl)-N’-ethylcarbodiimide hydrochloride (EDC)	Merk Life Science S.r.l.	Cat#03450
(sulfosuccinimidyl 6-[3′-(2-pyridyldithio)propionamido]hexanoate) (sulfo-LC-SPDP)	Covachem, LLC.	Cat# 13412
PEG-SH 550 Da	Biochempeg scientific inc.	Cat#20001-550
VIVIT peptide	Laura Marongiu et al. 2021, https://doi.org/10.1126/scisignal.aaz2120	N/A
H-Ferritin (HFn)	Tullio et al., (2021)https://doi.org/10.1021/acsabm.1c00724	N/A

**Critical commercial assays**		

IFN gamma Mouse ELISA Kit	Invitrogen	Cat #BMS606-2
CD4^+^ T Cell Isolation Kit, mouse	Miltenyi Biotec	Cat #130-104-454
RNeasy Mini Kit	Qiagen	Cat #74104
High-Capacity cDNA Reverse Transcription Kits	Applied Biosystems	Cat # 4368814

**Experimental models: Cell line**s		

A20	ATCC	TIB-208

**Experimental models: Organisms/strains**		

Mouse: C57BL/6	Charles River	C57BL/6NCrl
Mouse: B6.SJL-Ptprca Pepcb/BoyJ or B6 CD45.1	Jackson laboratory	002014
Mouse: Tg(KRT5-TFRC/OVA)1Ita or K5.mOVA	Jackson laboratory	No more available

**Oligonucleotides**		

Taqman probe: Ifng	Thermo Fisher scientific	Mm01168134_m1;
Taqman probe: Gapdh	Thermo Fisher scientific	Mm99999915_g1

### Resource availability

#### Lead contact

Further information and requests for resources and reagents should be directed to and will be fulfilled by the lead contact, (francesca.granucci@unimib.it).

#### Materials availability

This study did not generate new unique reagents.

### Experimental model and subject details

#### Cells

BMDCs were generated from bone marrow precursors of C57BL/6 WT, flushed from femurs, in Iscove’s modified Dulbecco’s medium (IMDM) (Euroclone) containing 10% heat-inactivated fetal bovine serum (Euroclone), 100 IU of penicillin, streptomycin (100μgmL^−1^), 2 mM l-glutamine (Euroclone), and granulocyte-macrophage colony-stimulating factor (GM-CSF) (10 to 20ngmL^–1^) for 8 days.

#### Mice

All mice were female and were used at 6 to 12 weeks of age. C57BL/6 WT CD45.2 mice, K5-mOVA and OT II transgenic mice were purchased from Charles River. NFATc2-deficient mice were provided by E. Serfling, Institute of Virology and Immunobiology, Wurzburg, Germany. DOG mice were provided by Natalio Garbi (University of Bonn, Germany). DEREG mice were obtained from T. Sparwasser, Twincore, Hannover, Germany. DEREG-OTII F1 mice were obtained by backcrossing DEREG and OTII mice for one generation. All animals were housed under pathogen-free conditions, and all experiments were carried out in accordance with relevant laws and institutional guidelines.

### Methods details

#### Antibodies and chemicals

The following antibodies used for flow cytometry were purchased from Biolegend: CD11bPE (clone M1/7), CD3Percp cy5.5 (clone 17A2), CD11c PEcy7 (clone N418) MHCII (clone AF-120.1), Ly6G pacific blue (clone 1A8), CD86 FITC, IL-2 FITC. TLR4-grade smooth LPS (*E*. *coli*, O55:B5) was purchased from Enzo Life Sciences and used *in vitro* at the concentration of 1 μg/mL. FK-506, thapsigargin, diphtheria toxin and SB-431542 poly[isobutylene-alt-maleic anhydride], dodecylamine, EDC and EDBE were purchased from Sigma-Aldrich. Sulfo-LC-SPDP and PEG-SH were acquired from Covachem, LLC. and Biochempeg Scientific Inc., respectively. VIVIT peptide suitable to be conjugated onto nanoparticles surface (CGGGKMAGPVIVITGPHEE) was assembled by conventional solid phase peptide synthesis (Fmoc-based protocols) (Marongiu et al., 2021), whereas HFn was produced as previously reported (Tullio et al., 2021).

#### Synthesis of MYTS-VIVIT and MYTS-PEG

The following protocol allowed for the preparation of 5 mg MYTS-VIVIT + 5 mg MYTS-PEG. To a round flask, 200 μL 0.5 M PMDA and 10 mg IONPs in 1 mL chloroform were added. The mixture was sonicated and then the solvent evaporated using a Rotavapor at 50°C. To the resulting film was resuspended in 10 mL of 0.5 M sodium borate buffer (SBB), pH 12, and sonicated until the suspension was clear. The solution was filtered using an Amicon Ultra Centrifugal filter with 50 kDa cut-off to dilute the buffer with water at 1/10k, and the material was quantified by UV absorption. Maintaining a 5–10mgmL^−1^ concentration, 50 mM EDBE (6 μL) and 0.1 M EDC (40 μL) were added to the solution and incubated at room temperature (RT) for 2 h. The sample was diluted to 1/100 using 20 mM SBB, pH 8.3, and filtered, next 5.1 mg sulfo-LC-SPDP (sSPDP) was added, and the mixture incubated 8 h at 4°C. The sample was filtered again using PBS to dilute excess reagents 1/10k. At this point, 0.1 mg MYTS were sampled and diluted to 0.5 mgmL^−1^. Functionalization with sSPDP was verified by UV absorption at 343 nm before and after adding DTT to 20 mM. The remainder of the sample was divided in two portions, 4.95 mg each. To one, 15 μL of 1gL^−1^ VIVIT and 8.5 μL of a 10gL^−1^ PEG-SH (550gmol^−1^) solutions were added. To the other prtion, PEG-SH only was added. Both batches were incubated at RT overnight. Next, the solutions were individually filtered to remove excess reagents.

#### Synthesis of PMDA-VIVIT NPs

For PMDA, the same procedure described for MYTS-VIVIT was followed, without adding IONPs. UV quantification for MYTS was done at 550 nm, whereas for PMDA UV absorption measurements were conducted at 250 nm.

#### HFn loading with VIVIT peptide

The VIVIT loading reaction was conducted exploiting HFn ability to disassemble/reassemble in response to pH variation. During this process, HFn randomly incorporate VIVIT peptides dissolved in the reaction buffer. Briefly, a 90-fold molar excess of VIVIT peptide was added to an HFn solution (1mgmL^−1^ in 0.15 M NaCl). Then, pH was adjusted to 2 by adding 0.1 M HCl and the solution was incubated 15 min at RT to allow HFn to completely disassemble. Next, the pH was slowly restored to physiological values (7.2–7.4) by adding 0.1 M NaOH. The solution was incubated at RT under stirring for 2 h. During this time HFn subunits spontaneously reassemble with a memory of their polymeric shape. After the incubation, the excess of VIVIT peptide was removed by washing the solution with PBS in 100 kDa Amicon centrifugal tubes. The amount of encapsulated VIVIT was calculated repeating the loading reaction with a rhodamine-conjugated peptide (λ_ecc_ 552; λ_em_ 575).

#### Transmission electron microscopy of NPs and biological samples

TEM analyses of NPs were conducted using a Zeiss EM109 instrument. Samples were prepared by evaporation of a drop of an aqueous NP solution deposited on a formvar/carbon-coated copper grid and air-dried. For cell culture analyses, cells were fixed in 2.5% glutaraldehyde in 0.1 M phosphate buffer, pH 7.2, for 2 h. After rinsing with the same phosphate buffer, samples were post-fixed in 1.5% osmium tetroxide for 2 h, dehydrated by 70, 90 and 100% ethanol, embedded in epoxy resin (PolyBed 812 Polysciences Inc., USA) and examined by TEM.

#### *In vitro* and *in vivo* nanoparticles uptake

BMDCs were incubated with MYTS-FITC or MYTS-PEG at 37°C or 4°C for 10, 30, 60 and 90 min. Cells were then washed with PBS and flow cytometric analyses were performed. For the distribution and uptake of NPs *in vivo*, mice were injected i.p. with NPs conjugated or not with FITC (100 μg/mouse). After 90 min, mice were euthanizing, and spleens, were collected and analyzed by flow cytometry. We identified DCs as CD11c++MHCII++ cells, macrophages as CD11b+ (and CD11c- Ly6G-) cells, neutrophils as Ly6G+CD11b+ cells, T as CD3^+^ cell and B as CD19^+^ cells.

#### ELISA assays

Concentration of IFN-γ in cell culture supernatants were assessed by ELISA kits purchased from Invitrogen.

#### *In vivo* treatment with NPs or FK-506

For *in vivo* administration, FK-506 was resuspended in 40% w/v HCO-60/ethanol at the dose of 40μgmL^−1^. MYTS-VIVIT and MYTS-PEG were diluted in sterile PBS at 100μgmL^−1^. Mice were injected i.p. with FK-506 or NPs the day before the transplant. FK506 and NPs were injected every other day until the end of the treatment as specified in the Figures. For allograft experiments (Figure 6C) FK506 was injected every day until the end of the treatment as specified in the Figure.

#### Skin grafting

##### Minor and major histocompatibility antigen mismatch

Recipient animals (female C57BL/6 mice) were shaved on the dorsum 24 h before skin grafting. Recipient mice were then anesthetized and a section of donor skin from tail of WT, NFATc2-deficient, or DOG mice with minor or major histocompatibility mismatch, or from WT mice with major histocompatibility mismatch was transplanted onto the dorsum of recipient mice.

##### Transplantation of OVA-expressing skin

DEREG-OTII CD45.1 mice were injected with diphtheria toxin, 40 ng/g for 3 days to deplete Foxp3^+^Treg cells. Mice were then euthanized and CD4^+^ DEREG-OTII (FOXp3^-^) cells were isolated with CD4^+^ T Cell Isolation Kit (Miltenyi) and infused I.V. into C57Bl/6 recipients (3 × 10ˆ5 per mouse). Two hours later, recipients were transplanted with tail skin from K5-mOVA donors.

Recipients were treated with MYTS-VIVIT or MYTS-PEG NPs (100μg/mouse) or FK506 (40μg/mouse for all conditions) every other day starting from day− 1 through day 50 after transplantation. For allotransplants (Figure 6C) FK506 was used at 100μg/mouse and was injected every day started from day −1.

Rejection was monitored after dressing removal at day 14 after surgery as described in Figures 6A and 6B. Treatments were stopped on day 50 and rejection was monitored after an additional 20 days (day 70 after surgery).

Ulceration, presence of crusts, and desquamation were considered the endpoints of rejection (Figure S6).

#### T cell activation *in vivo* in NP treated animals

OTII mice were treated with MYTS-VIVIT or MYTS-PEG NPs (100ug/mouse) every other day or FK506 (40μg/mouse) every day for two weeks. At the end of the treatment, syngeneic BMDCs, pre-activated with LPS (1ug/mL O/N) and pulsed with Ova (10uM for 2 h), were injected in the footpad of treated OTII mice or untreated mice as control. After 72h, popliteal lymph nodes were collected to assess the activation of T cells by measuring IL-2 production by intracellular staining.

#### Immunofluorescent staining

A20 cells expressing the NFAT4-GFP fusion protein (100.000) were plated on glass coverslips. Cells were then incubated with MYTS-VIVIT, MYTS-PEG (25μgmL^−1^) or FK-506 (10ngmL^−1^) for 90 min and then stimulated with LPS (1μgmL^−1^) plus thapsigargin (50 nM) for 40 min. After the stimulation, A20 cells were fixed in paraformaldehyde 4%. All samples were mounted in FluorSave™ Reagent (Calbiochem) and were imaged by Leica TCS SP2 confocal microscope. ImageJ software was used for image analysis and processing.

#### Gene expression analysis

Grafted skins were collected, gently washed in cold PBS, lysed with TRIzol reagent and mechanically disrupted using a TissueLyser (20 shakes/sec for 10 min). Total RNA was extracted using Qiagen RNeasy Mini Kit (catalog no. 74104). Single-strand complementary DNA (cDNA) was synthesized using High-Capacity cDNA Reverse Transcription Kits (catalog no. 4368814, Applied Biosystems). The NanoDrop (Thermo Scientific) was used to titer mRNA, and amplification was performed using the TaqMan Gene Expression Master Mix (catalog no. 4369016, Applied Biosystems) and TaqMan probes (Ifng, Mm01168134_m1; Gapdh Mm99999915_g1) in a 7500 Fast Real-Time PCR System (Applied Biosystems) and finally relative mRNA expression was calculated using the ΔC_t._

#### Dendritic cells migration assay

Mice were anesthetized and an FITC-based cream was gently applied at the basal of the tail. After 18 h, the mice were euthanized, the draining inguinal lymph nodes were collected and smashed and the cells suspension obtained were incubated with anti CD11c PE, anti-MHCII APC antibodies for 20 min at 4°C. Samples were analyzed with flow cytometry.

#### Ethical approvals

Animal studies have been performed after the approval from the Italian Ministry of Health, and the animals’ care was in accordance with institutional guidelines.

#### Statistical analysis

Means were compared by either unpaired parametric *t* tests or two-way analysis of variance (ANOVA). Data are expressed and plotted as means ± squared deviations from the mean (SDM) or ±SEM values. Sample sizes for each experimental condition are provided in the figure legends. *p* values for Kaplan-Meier curves were calculated with log-rank test. All *p* values were calculated using Prism (GraphPad). Differences were considered significant if p ≤ 0.05.

## Data Availability

• All data reported in this paper will be shared by the lead contact on request. • This article does not report original code.
