# Supplementary material for: Specific immunosuppressive role of nanodrugs targeting calcineurin in innate myeloid cells
Source: iScience. 2022 Aug 30;25(10):105042. doi: 10.1016/j.isci.2022.105042 (PMC9482116; doi:10.1016/j.isci.2022.105042)

iScience, Volume *25*

Supplemental information

Specific immunosuppressive role of nanodrugs targeting calcineurin in innate myeloid cells

Miriam Colombo, Laura Marongiu, Francesca Mingozzi, Roberta Marzi, Clara Cigni, Fabio Alessandro Facchini, Rany Rotem, Mihai Valache, Giulia Stucchi, Giuseppe Rocca, Laura Gornati, Maria Antonietta Rizzuto, Lucia Salvioni, Ivan Zanoni, Alessandro Gori, Davide Prosperi, and Francesca Granucci

Supplementary Figure S1. DC depletion from DOG mice, related to Figure 1B

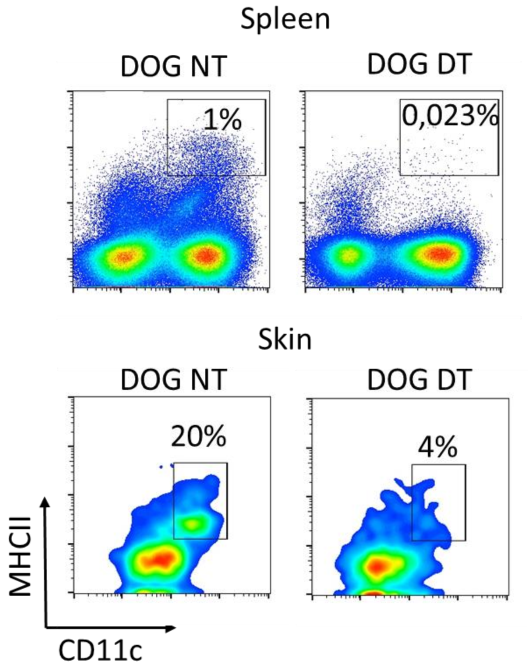

Supplementary Figure S2. Flow cytometric analysis of activation markers expression by NFATC2-deficient and -sufficient BMDCs after 8 days of in vitro culture and activation with LPS, related to Figure 1D

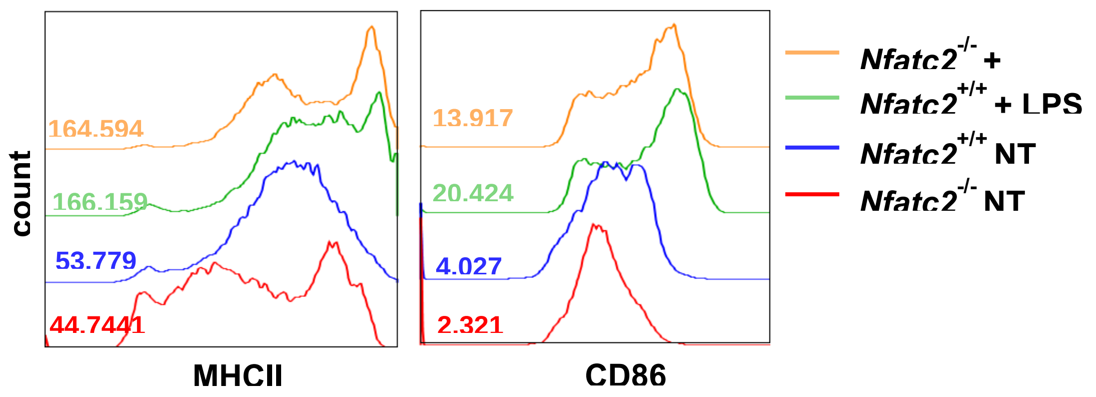

Supplementary Figure S3. A-C VIVIT nanoparticles. Representative transmission electron micrographs and schematic representation (insets) of (A) MYTS-VIVIT; (B) PMDA-VIVIT, and (C) HFn(VIVIT), related to Figure 2.

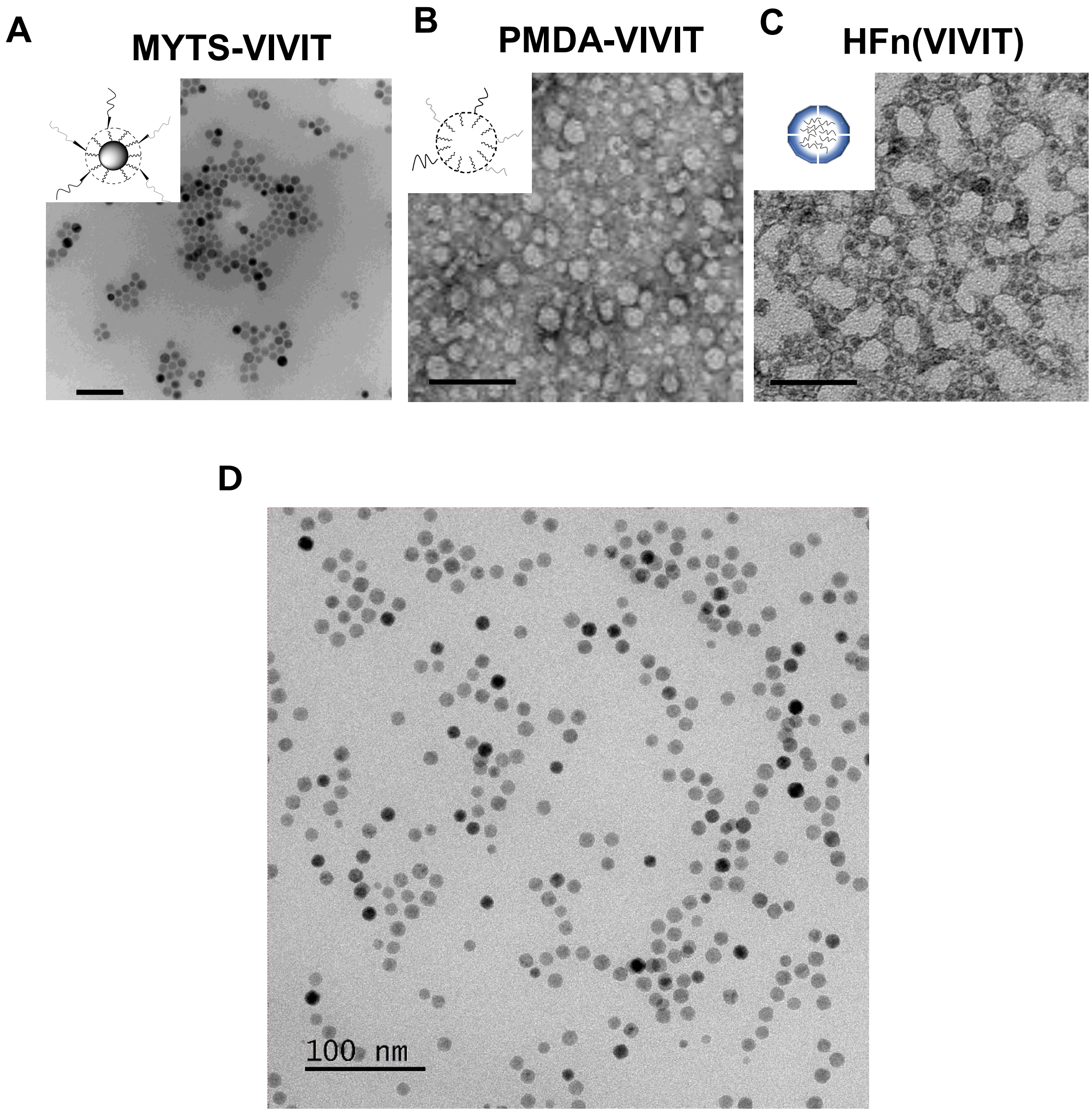

Supplementary Figure S4. (A) Synthesis of PMDA. (B) Synthesis of MYTS-VIVIT. (C) Synthesis of PMDA-VIVIT. (D) Synthesis of HFn(VIVIT) from recombinant H-Ferritin, related to star methods.

A)

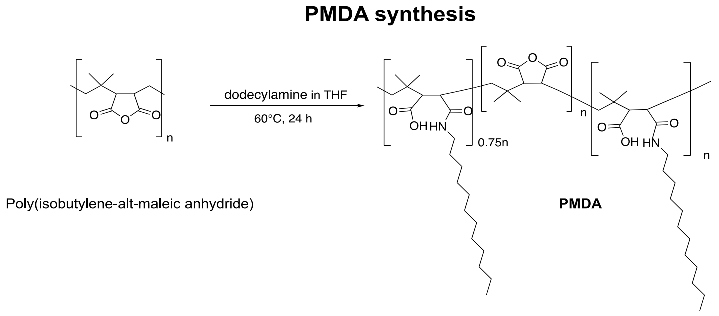

B)

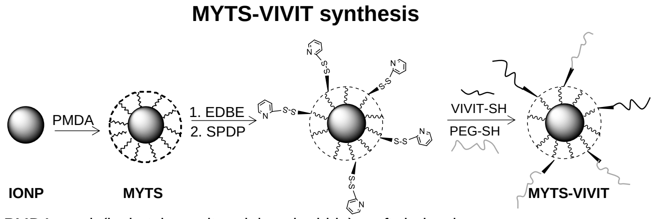

PMDA = poly(isobutylene-alt-maleic anhydride)-graft-dodecyl

EDBE = 2,20-(ethylenedioxy)bis(ethylamine)

SPDP = N-succinimidyl-3-[2-pyridyldithio] propionate

VIVIT = NH2-**C**GGGKMAGPPHI**VIVIT**GPHVI-COOH

C)

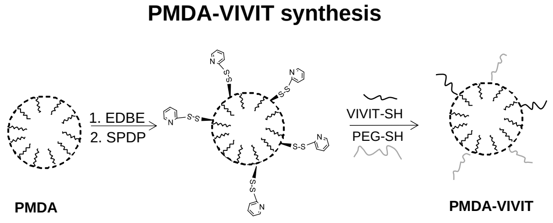

D)

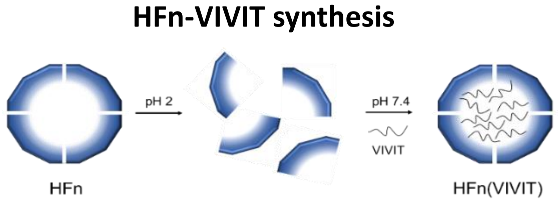

Supplementary Figure S5. Endosomal escape of MYTS in DCs, related to Figure 4.

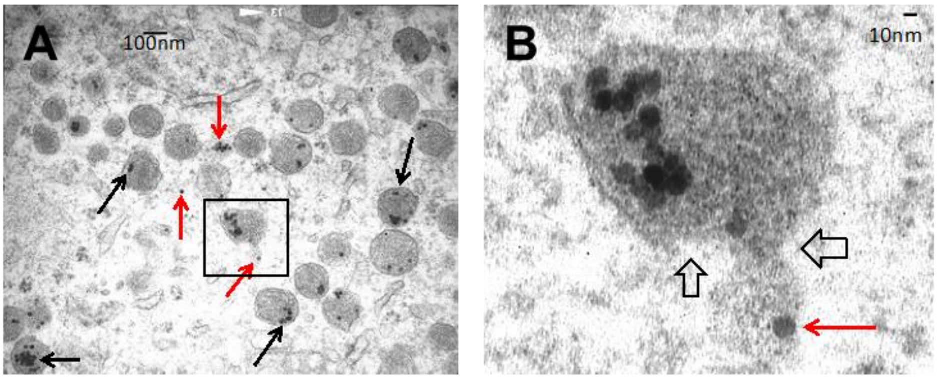

Supplementary Figure S6. Examples of rejected (presence of crusts) and accepted transplants, related to Figure 1A, 1B and 6.

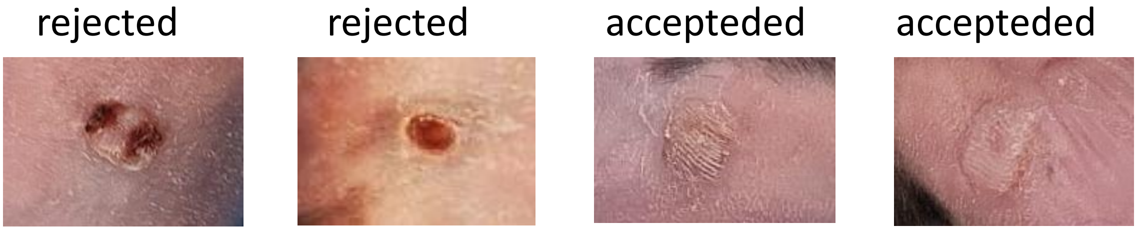

Supplement: Document S1. Figures S1–S6 [file mmc1.pdf]
